# Cinnamtannin A2 protects the renal injury by attenuates the altered expression of kidney injury molecule 1 (KIM-1) and neutrophil gelatinase-associated lipocalin (NGAL) expression in 5/6 nephrectomized rat model

**DOI:** 10.1186/s13568-020-01022-6

**Published:** 2020-05-08

**Authors:** Na Li, Mingzhu Xu, Mei Wu, Guanjie Zhao

**Affiliations:** 1 Department of Nephrology, The third Hospital of Jilin University, No 126 Xiantai Street, Changchun, 130033 Jilin People’s Republic of China; 2 Central Laboratory, The third Hospital of Jilin University, Changchun, 130033 Jilin People’s Republic of China

**Keywords:** Cinnamtannin A2, Nephractomy, Oxidative stress, Cytokines, Microalbuminurea

## Abstract

Present investigation determines the protective effect of Cinnamtannin A2 against chronic renal failure (CRF). 5/6 nephrectomized rat model was used to induced CRF by removing the kidneys and rats were treated with Cinnamtannin A2 10 mg/kg, i.p. for the period 30 days. Nephroprotective effect Cinnamtannin A2 was assessed by estimating the biochemical parameters of renal function test and cytokines in the serum of nephractomized rats. Oxidative stress parameters were estimated in the kidney tissue and western blot assay and qRT-PCR assay was performed to determine the expression of protein in renal tissue of nephractomized rats. Moreover histopathology study was done to observe the tubular injury. Data of the report reveals that treatment with Cinnamtannin A2 ameliorates the altered level of creatinine, blood urea nitrogen (BUN), Neutrophil gelatinase-associated lipocalin (NGAL), Kidney Injury Molecule-1 (KIM-1) and cytokines in the serum and microalbuminurea in the urine of 5/6 nephrectomized rat. Oxidative stress level was reduced in Cinnamtannin A2 treated group than CRF group. Moreover treatment with Cinnamtannin A2 attenuates the altered expression of proteins involved in Nrf2-Keap1 pathway in the kidney tissue of 5/6 nephrectomized rat. Result of histopathology reveals that tubular injury score was reduced in the kidney tissue of Cinnamtannin A2 treated group than CRF group. In conclusion, data of the report suggest that treatment with Cinnamtannin A2 ameliorates the level of KIM1 and NAGL in 5/6 nephractomized rats by regulating Nrf2- Keap1 pathway.

## Introduction

Chronic renal failure (CRF) is one of the major causes of cardiovascular complication and mortality throughout the globe. Renal failure is commonly characterized by reduction of renal function, scar to tubulointerstitial and glomeruli, vascular modeling, oxidative stress and chronic inflammation (Imig and Ryan 2013). There are several rodent models reported for its experimental renal failure study. Literature reveals that pathological changes occur clinically in chronic renal failure resembles with the partially nephrectomized rat model (Nogueira et al. 2017). There are several pathway involved in the development and progression renal failure including inflammatory and oxidative stress pathway such as impairment of Nrf2-Keap1 pathway (David et al. 2017). Some novel biomarkers such as kidney injury molecule 1 (KIM-1) and neutrophil gelatinase-associated lipocalin (NGAL) were reported to be altered in chronic renal failure (Spasojević-Dimitrijeva et al. 2017). Conventional drug available for the treatment of renal failure has several limitations and thus there is a need to some new molecules for the management of it.

Last from few decades alternative medicine has shown potential for the management of renal failure and complication associated with it. Cinnamtannin A2 is chemically a procyanidin isolated from several sources such as pine bark, immature apples, red wine and chocolate (Wei et al. 2011). Cinnamtannin A2 has potential anti diabetic activity by increasing insulin secretion and GLP-1 (Yoko et al. 2013). Moreover it upregulates the expression of corticotrophin releasing hormone (CRH) and CRH level was reported to be reduced in the chronic renal failure patients (Quintanar and Guzmán-Soto 2013). Thus present report evaluates the nephroprotective effect of Cinnamtannin A2 against CRF.

## Materials and methods

### Animal

Male Sprague–Dawley rats weighing 250–300 g were kept under a 12-h light/dark cycle at 60 ± 5% humidity and 24 ± 3 °C. Protocols used in the animals were approved by Institutional Animal Ethical Committee of China–Japan Union Hospital of Jilin University, China (IAEC/DJUH/JU/2018/29).

### Chemicals

Cinnamtannin A2 was procured from Sigma Aldrich Pvt Ltd, USA and enzyme linked immunosorbent assay (ELISA) kits for NGAL, KIM-1, cystatin C, interleukin (IL)-1β, IL-6, and NF-kB were purchased from ThermoFisher Scientific, USA. All primary antibodies used in the Western blot assays were procured from Cell Signaling Technology, China.

### Experimental

All the animals were anesthetized by i.p. administration of chloral hydrate at a dose of 33 mg/100 g body weight. Animals were subjected for 5/6 nephrectomy by removing entire right kidney and upper and lower poles of the left kidney as per previously reported studies. Animals were divided into three different groups such as Sham group; CRF group and Cinnamtannin A2 group receives Cinnamtannin A2 10 mg/kg, i.p. for the period 30 days after the surgery.

#### Determination of biochemical parameters of renal function

Blood samples were collected from each animal at on 0, 7, 20 and 30th day of protocol and serum was separated from it. Level of BUN and Creatinine was estimated in the serum at different time interval of the protocol as per the direction given by the manufacturer of the kits. Moreover level of microalbumin in the urine was also observed by using metabolic cage for the collection of urine.

#### Enzyme-linked immunosorbent assay

ELISA method was used to determine the level of kidney injury molecule 1 (KIM-1) and neutrophil gelatinase-associated lipocalin (NGAL), cystatin C, IL-1β, IL-6 and NF-kB in the serum of CRF rats as per the direction given by the manufacturer of ELISA kit.

#### Determination of oxidative stress

Malondialdehyde (MDA), nitric oxide (NO) and glutathione (GSH) levels and catalase (CAT) and superoxide dismutase (SOD) activities were estimated in kidney tissues using ELISA kits according to the manufacturer’s instructions.

#### Western blot assays

Total protein extraction from the kidney tissues was accomplished by lysing the tissues with a solution of 150 mM NaCl, 50 mM Tris–HCl, NP40 protein lysis buffer, and 5 mM ethylene-diaminetetraacetic acid (EDTA; pH 8.0), supplemented with a protease inhibitor cocktail. The protein lysates were centrifuged for 10 min at 13,400 rpm, and the supernatants were collected for further examination. The DC Protein Assay was performed to estimate the total protein concentration. The isolated proteins were separated by 10% sodium dodecyl sulphate–polyacrylamide gel electrophoresis and then transferred to polyvinylidene difluoride membranes, and the membranes were blocked in 5% fresh non–fat dry milk. The membranes were incubated overnight at 4 °C with primary antibodies against IkBα (1:500), Keap1 (1:200), Nrf2 (1:100), p-38 (1:200), NF-κB (1:100), p-NF-κB (1:100), and β-actin (1:100). Subsequently, the membranes were incubated in secondary antibodies at room temperature for 60 min. The blots were visualized by chemiluminescence, and densitometric analysis of the protein bands was performed using ImageLab software.

#### qRT-PCR

Trizol Reagent was used to isolate the total RNA from kidney tissues as per the directions given by the manufacturer. Reverse transcription kit was used to reversely transcribed cDNA from RNA as per the instruction given by the manufacturer of kit. ABI Prism 7500 system was used to take SYBR green/fluorescein qPCR Master Mix kit. The conditions were as follows: 50 °C for 2 min; 95 °C for 10 min; and 40 cycles of 95 °C for 30 s and 60 °C for 30 s. The resulting data were analyzed using the comparative Ct method (2-^ΔΔ^Ct).

### Primer forward reverse

KIM-1 5′-AACGCAGCGATTGTGCATCC-3′ 5′-GTACACTCACCATGGTAACC-3′

NGAL 5′-GATGAACTGAAGGAGCGATTC-3′ 5′-TCGGTGGGAACAGAGAAAAC-3′

IL-6 5′-GACTGATGCTGGTGACAACC-3′ 5′-GCCATTGCACAACTCTTTTC-3′

NF-*κ*B 5′-GTATGGCTTCCCGCACTATGG-3′ 5′-TCGTCACTCTTGGCACAATCTC-3′ MCP-1 5′-GTGTCCCAAAGAAGCTGTAGTATTT-3′ 5′-GTGCTGAAGTCCTTAGGGTTGA-3′ GAPDH 5′-GGAAAGCTGTGGCGTGAT-3′ 5′-AAGGTGGAAGAATGGGAGTT-3′

#### Determination of histopathological changes

Isolated kidneys were fixed in 10% formalin for 1 day at room temperature, and a standard protocol was performed to prepare the histological slides. Briefly, the kidney tissues were dehydrated with ethanol and then seeded into liquid paraffin. Next, a wax cube of the kidney sample was prepared, and 4-µm thick slices of renal tissue were sectioned using a microtome. The tissue sections were then subjected to the Jones’ periodic acid–Schiff (PAS) and alterations of the histopathological changes in the kidney tissues were evaluated using Olympus BX50 bright field microscope.

### Statistical analysis

All data are shown as mean ± standard deviation (SD) (n = 10). Results were compared using one-way analysis of variance and the Dunnett post hoc test (GraphPad Prism ver. 6.1 software; GraphPad, La Jolla, CA, USA). P values < 0.05 were considered to be significant.

## Results

### Effect of Cinnamtannin A2 on renal function

**Fig. 1:**
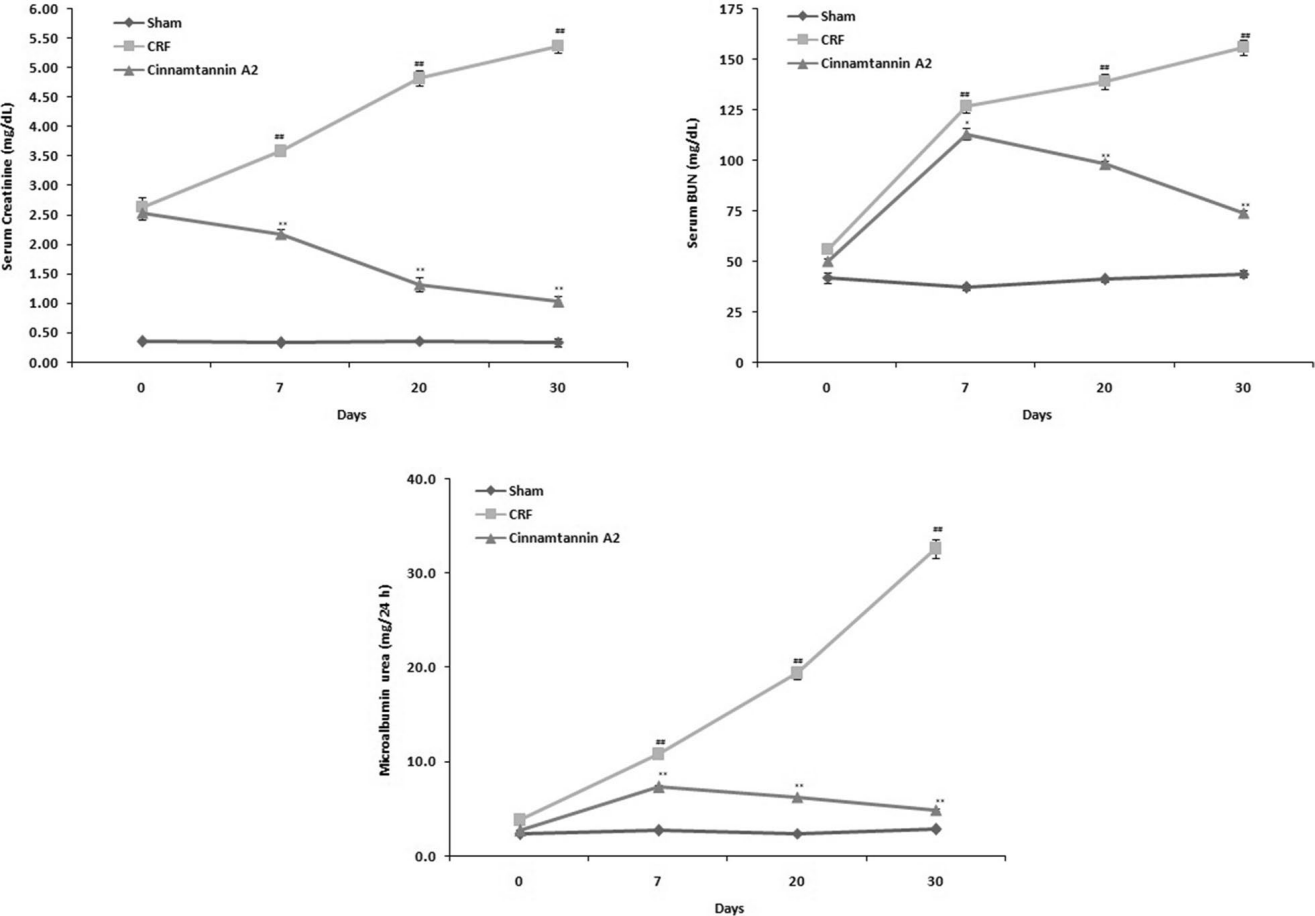
Effect of Cinnamtannin A2 on the level of biochemical parameter in the serum and urine of 5/6 nephractomized rat. Mean ± SD (n = 10); ^##^p < 0.01 than Sham operated group; **p < 0.01 than CRF group

Biochemical parameters for the assessment of renal function was assessed in the serum and urine of Cinnamtannin A2 treated 5/6 nephractomized rats. change in the normal level of serum creatinine and BUN and microalbumin urea in the urine confirms the renal dysfunction and data of the study also supports it. Cinnamtannin A2 treatment for the period of 30 days ameliorates the altered level of serum creatinine and BUN and microalbumin urea in the urine of 5/6 nephroactomized rats (Fig. 1).

### Effect of Cinnamtannin A2 on the mediators of inflammation

**Fig. 2:**
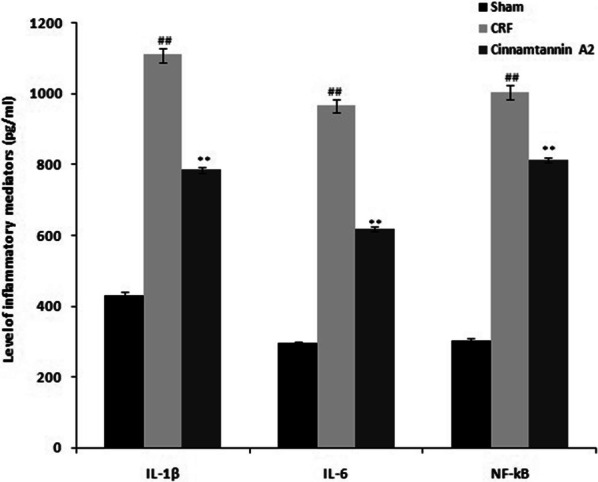
Effect of Cinnamtannin A2 on the level of cytokines in the serum of 5/6 nephractomized rats. Mean ± SD (n = 10); ^##^p < 0.01 than Sham operated group; **p < 0.01 than CRF group

Mediators of inflammation level was determined in the serum of Cinnamtannin A2 treated 5/6 nephractomized rats as shown in Fig. 2. Level of IL-1β, IL-6 and NF-kB was significantly (p < 0.01) enhanced in the serum of CRF group than sham operated group. However treatment with Cinnamtannin A2 reduces the level of cytokines in the serum of 5/6 nephractomized rats compared to CRF group.

### Effect of Cinnamtannin A2 on the biochemical parameters

**Fig. 3:**
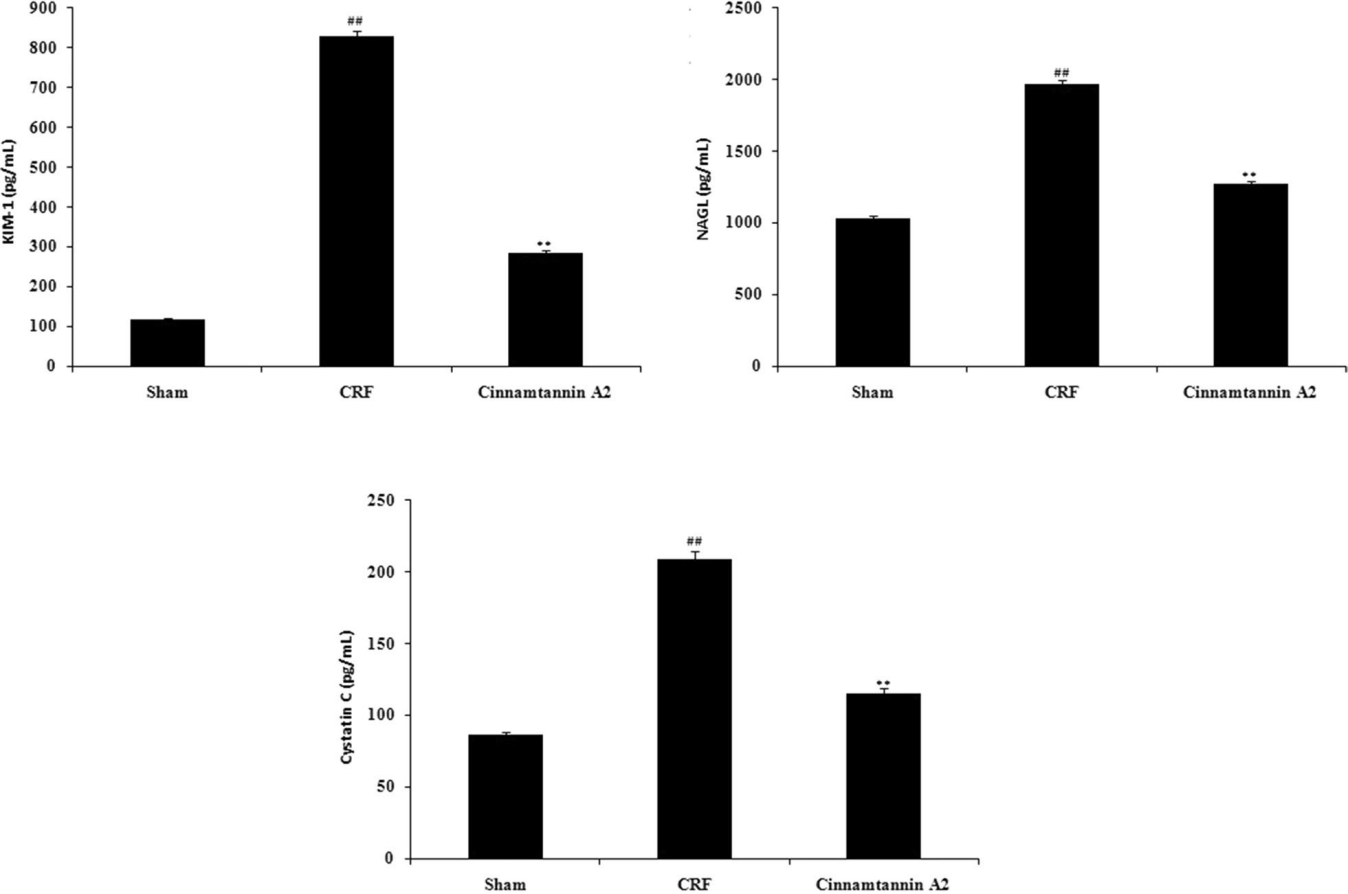
Effect of Cinnamtannin A2 on the level of biochemical parameters in the serum of 5/6 nephractomized rats. Mean ± SD (n = 10); ^##^p < 0.01 than Sham operated group; **p < 0.01 than CRF group

Figure 3 shows the effect of Cinnamtannin A2 on the biochemical parameters such as NAGL, KIM-1 and cystatin C in the serum of nephractomized rats by using ELISA. There was increase in the level of NAGL, KIM-1 and cystatin C in the serum of CRF group than sham perated group. Level of NAGL, KIM-1 and cystatin C was found to be significantly reduced (p < 0.01) in the serum of Cinnamtannin A2 treated group than CRF group.

### Effect of Cinnamtannin A2 on the oxidative stress parameters

**Fig. 4:**
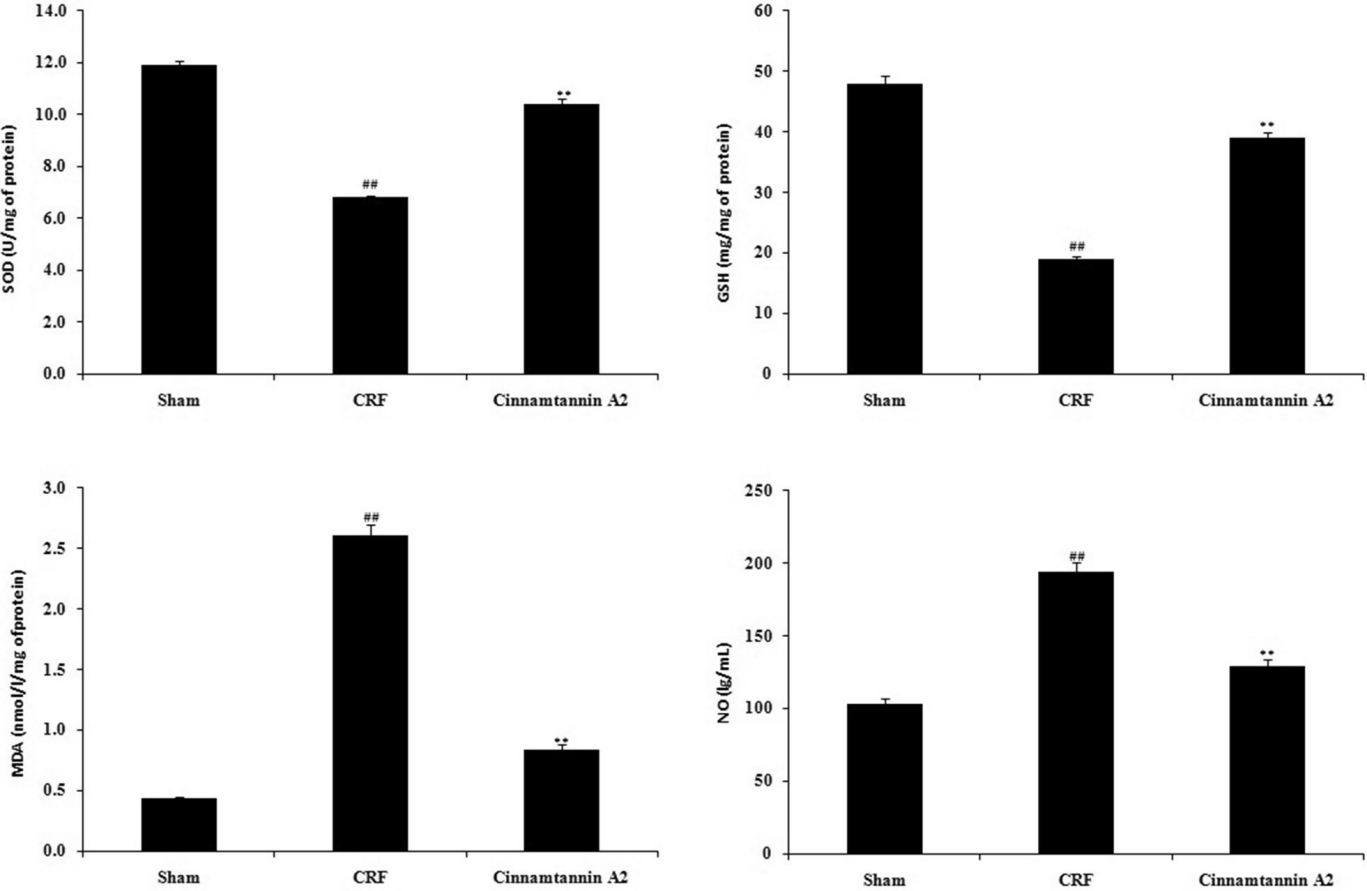
Effect of Cinnamtannin A2 on the level of parameters of oxidative stress in the kidney tissue of 5/6 nephractomized rats. Mean ± SD (n = 10); ^##^p < 0.01 than Sham operated group; **p < 0.01 than CRF group

Parameters of oxidative stress such as level of NO, GSH and MDA and activity of SOD was assessed in the kidney tissue of Cinnamtannin A2 treated 5/6 nephractomized rats as shown in Fig. 4. Level of GSH was reduced and level of MDA and NO was enhanced in the kidney tissue of CRF group than sham operated group. Moreover activity of SOD was reduced in the kidney tissue of CRF group than sham group. There was reduction in the level of NO and MDA and increase in the level of GSH in Cinnamtannin A2 treated group than CRF group. Activity of SOD was enhanced in the kidney tissue of Cinnamtannin A2 treated group than CRF group.

### Effect of Cinnamtannin A2 on the mRNA expression of NAGL, KIM-1, NF-kB, IL-6 and MCP-1

**Fig. 5:**
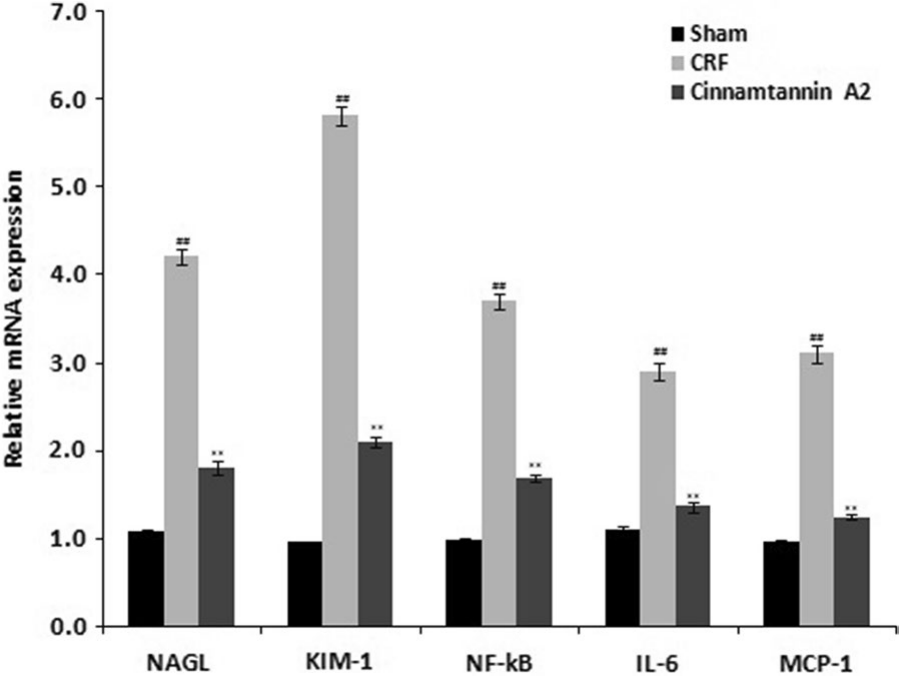
Effect of Cinnamtannin A2 on the mRNA expression of NAGL, KIM-1, NF-kB, IL-6 and MCP-1 in the kidney tissue of 5/6 nephractomized rats. Mean ± SD (n = 10); ^##^p < 0.01 than Sham operated group; **p < 0.01 than CRF group

qRT-PCR method was used to determine the mRNA expression NAGL, KIM-1, NF-kB, IL-6 and MCP-1 in the kidney tissue of 5/6 nephractomized rats. mRNA expression of NAGL, KIM-1, NF-kB, IL-6 and MCP-1 was enhanced in the kidney tissue of CRF group than sham operated group. There was reduction in the mRNA expression NAGL, KIM-1, NF-kB, IL-6 and MCP-1 in the kidney tissue of Cinnamtannin A2 treated group than CRF group (Fig. 5).

### Effect of Cinnamtannin A2 on the expression of IkBα, Keap1, Nrf2, p-38 and p-NF-κB proteins

**Fig. 6:**
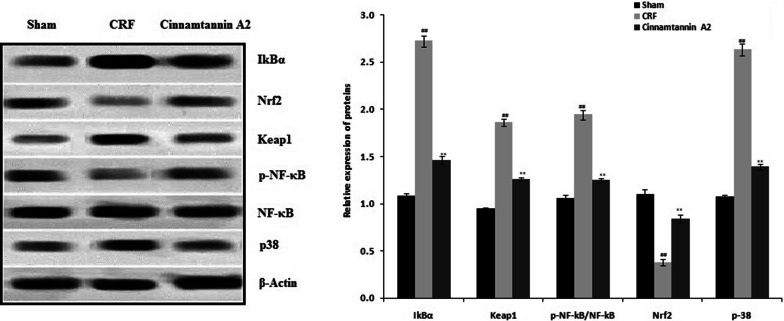
Effect of Cinnamtannin A2 on the expression of IkBα, Keap1, Nrf2, p-38 and p-NF-κB proteins in the kidney tissue of 5/6 nephractomized rats. Mean ± SD (n = 10); ^##^p < 0.01 than Sham operated group; **p < 0.01 than CRF group

Expression of IkBα, Keap1, Nrf2, p-38 and p-NF-κB protein was determined in the kidney tissue of nephractomized rats by western blot assay (Fig. 6.). There was increase in the expression of IkBα, Keap1, p-38 and p-NF-κB proteins and decrease in the expression of Nrf2 proteins in the kidney tissue of CRF group than sham operated group. Treatment with Cinnamtannin A2 ameliorates the altered expression of IkBα, Keap1, Nrf2, p-38 and p-NF-κB proteins in the kidney tissue of 5/6 nephractomized rats.

### Effect of Cinnamtannin A2 on the histopathology of kidney tissue

**Fig. 7:**
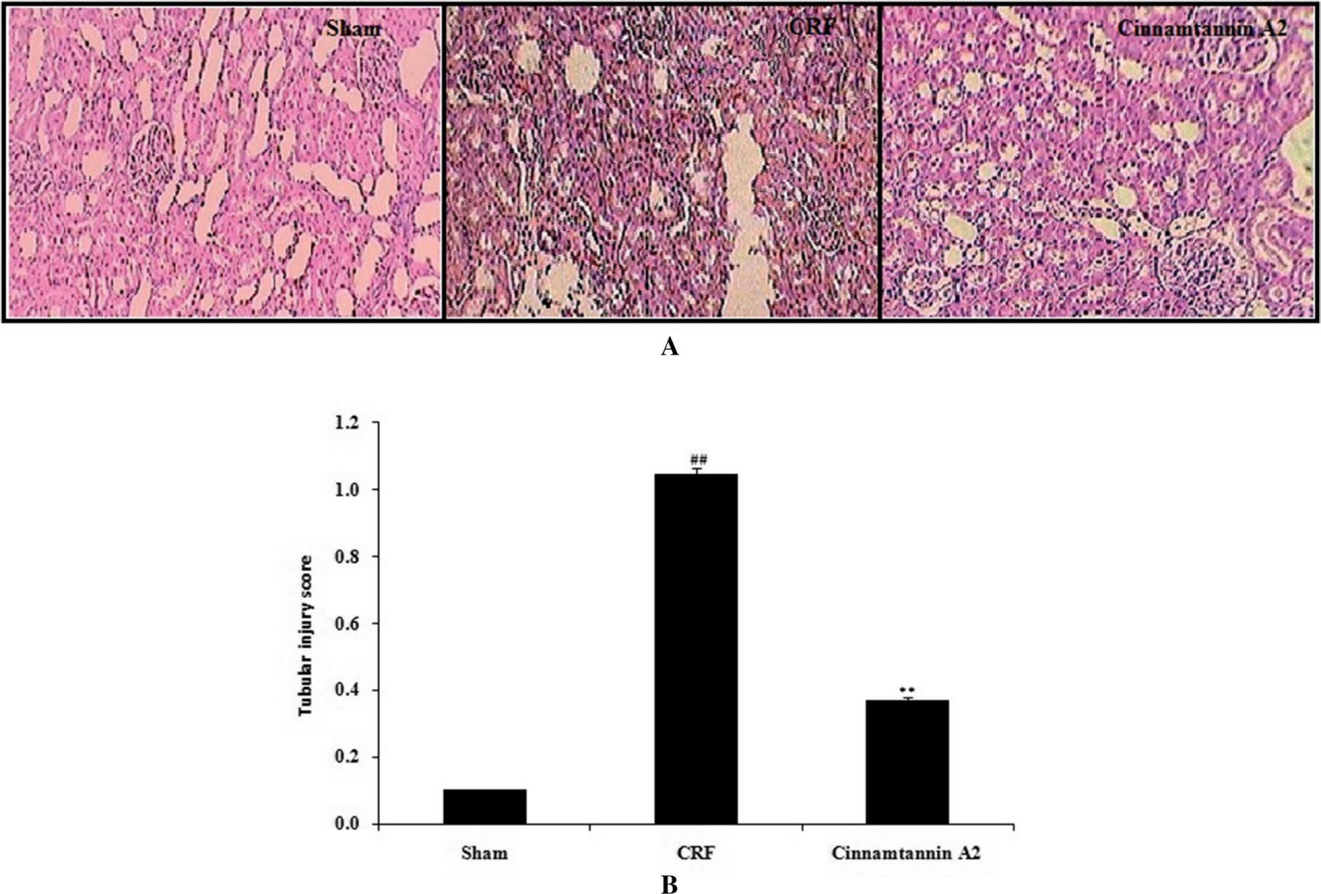
Effect of Cinnamtannin A2 on the histopathology of kidney tissue of 5/6 nephractomized rats. **a** PAS staining to the TS of kidney tissue. **b** Tubular Injury score. Mean ± SD (n = 10); ^##^p < 0.01 than Sham operated group; **p < 0.01 than CRF group

Effect of Cinnamtannin A2 was assessed on the histopathology of kidney tissue of 5/6 nephractomized rats by PAS staining as shown in Fig. 7. There was increase in the tubular injury score in CRF group than sham operated group. There was reduction in the tubular injury score in the Cinnamtannin A2 treated kidney tissue of 5/6 nephractomized rats.

## Discussion

Chronic renal failure is one of the major causes of cardiovascular disorders and death throughout the globe. Conventional drug used for the management of renal failure and associated complication has several limitations. Present investigation determines the protective effect of Cinnamtannin A2 against chronic renal failure. Nephroprotective effect Cinnamtannin A2 was assessed by estimating the biochemical parameters of renal function test and cytokines in the serum of nephractomized rats. Oxidative stress parameters were estimated in the kidney tissue and western blot assay and qRT-PCR assay was performed to determine the expression of protein in renal tissue of nephractomized rats. Moreover histopathology study was done to observe the tubular injury.

In chronic renal failure patients GFR decrease due to injury to nephron and development of it assessed by microalbuminurea (Levin 2013). There are several animal models of CRF stabilized but 5/6 nephractomized rat model resembles the characteristics of renal failure clinically (Bao et al. 2018). Present investigation also performed the study on the 5/6 nephractomized rat model and data of the study confirms the confirmation of CRF in rats. Literature reveals that KIM-1 and NGAL are the novel and more sensitive biomarkers renal function, which enhances in renal dysfunction and report of study also confirms it (Lopez-Giacoman and Madero 2015). Result of the study reveals that treatment with Cinnamtannin A2 ameliorates the alter level of biochemical parameters of renal function in serum and urine of 5/6 nephractomized rats.

There are several factors including oxidative stress contributes in the development of renal failure (Sung et al. 2013). Oxidative stress reported to be enhanced in the kidney tissue of renal injured rats (Tripathi et al. 2014) and data of the presented report reveals that treatment with Cinnamtannin A2 attenuates the altered parameters of oxidative stress. Inflammatory cytokines such as IL-6, IL-1β and NF-kB also reported contributes in the development of renal failure (Imig and Ryan 2013) and level of them was found to ameliorated in Cinnamtannin A2 treated 5/6 nephractomized rats.

Literature suggests that in CRF expression of Keap1 elevated by the activation of Nrf2 occurs due to inflammation and oxidative stress (Arellano-Buendía et al. 2016). In kidney injury oxidative stress and inflammatory cytokines reported to be enhanced which also altered the Nrf2-Keap1 pathway in the kidney tissue (Rapa et al. 2019). Literature reported that in Nrf2 knockout mice level of cytokines and oxidative stress in the kidney tissue (Ruiz et al. 2013). Data of the study suggest that treatment with Cinnamtannin A2 ameliorates the altered expression of IkBα, Keap1, Nrf2, p-38 and p-NF-κB proteins in the kidney tissue of 5/6 nephractomized rats.

In conclusion, data of the report suggest that treatment with Cinnamtannin A2 ameliorates the level of KIM1 and NAGL in 5/6 nephractomized rats by regulating Nrf2- Keap1 pathway.

## Data Availability

The supporting data for present fndings is under ethics restrictions and is hence not presented here.
