# Correction: Cinnamtannin A2 protects the renal injury by attenuates the altered expression of kidney injury molecule 1 (KIM-1) and neutrophil gelatinase-associated lipocalin (NGAL) expression in 5/6 nephrectomized rat model

**DOI:** 10.1186/s13568-022-01502-x

**Published:** 2023-02-06

**Authors:** Na Li, Mingzhu Xu, Mei Wu, Guanjie Zhao

**Affiliations:** 1 Department of Nephrology, The Third Hospital of Jilin University, No 126 Xiantai Street, Changchun, 130033 Jilin People’s Republic of China; 2 Central Laboratory, The Third Hospital of Jilin University, Changchun, 130033 Jilin People’s Republic of China

**Correction: AMB Expr (2020) 10:87** 10.1186/s13568-020-01022-6

Following publication of the original article (Li et al. 2022), the author noticed the errors in the figures. In the published version, Figs. 6 and 7A are misused. The author has wrongly uploaded these figures in the manuscript package which have been processed by the typesetter. However, the text citations and captions of these figures seem to be correct. The corrected Figs. 6 and 7A have been published in this correction.

**Fig. 6:**
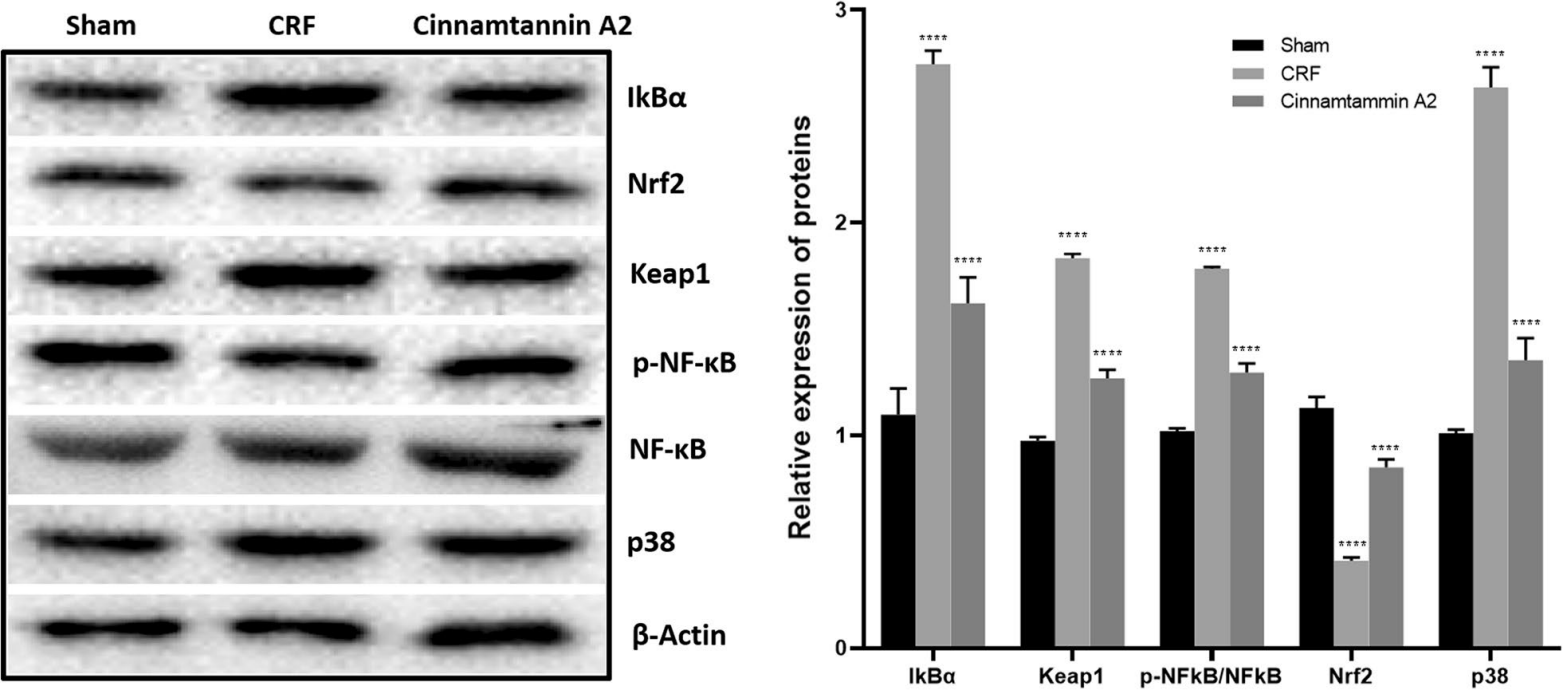
Effect of Cinnamtannin A2 on the expression of IkBα, Keap1, Nrf2, p-38 and p-NF-κB proteins in the kidney tissue of 5/6 nephractomized rats. Mean ± SD (n = 10); ^##^p < 0.01 than Sham operated group; **p < 0.01 than CRF group

**Fig. 7:**
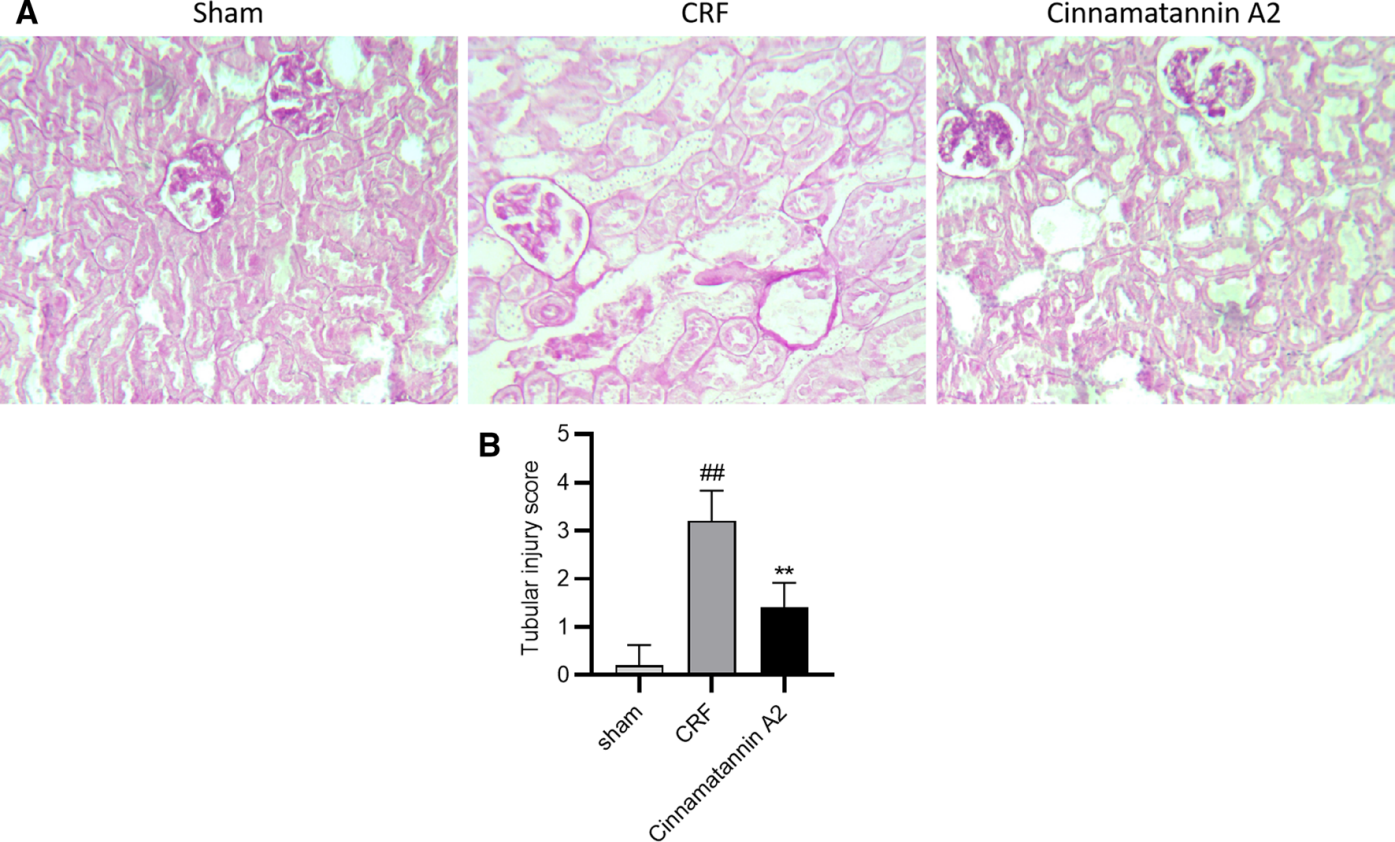
Effect of Cinnamtannin A2 on the histopathology of kidney tissue of 5/6 nephractomized rats. **a** PAS staining to the TS of kidney tissue. **b** Tubular Injury score. Mean ± SD (n = 10); ^##^p < 0.01 than Sham operated group; **p < 0.01 than CRF group

## References

[CR1] Li N, Xu M, Wu M, Zhao G (2020). Cinnamtannin A2 protects the renal injury by attenuates the altered expression of kidney injury molecule 1 (KIM-1) and neutrophil gelatinase-associated lipocalin (NGAL) expression in 5/6 nephrectomized rat model. AMB Expr.

